# Evaluation of perioperative lung ultrasound scores in robotic radical prostatectomy: prospective observational study

**DOI:** 10.1007/s11701-025-02272-x

**Published:** 2025-03-11

**Authors:** İrem Vuran Yaz, Emre Sertaç Bingül, Mert Canbaz, Evren Aygün, Mehmet Öner Şanlı, Faruk Özcan, Meltem Savran Karadeniz

**Affiliations:** 1 Department of Anesthesiology and Reanimation, Istanbul University Istanbul Faculty of Medicine, Millet Cd. Cerrahi Monoblok Giriş Kat, 34093 Fatih, Istanbul Turkey; 2 Department of Anesthesiology, Eyupsultan State Hospital, Istanbul, Turkey; 3 Department of Anesthesiology, Liv Vadistanbul Hospital, Istanbul, Turkey; 4 Department of Urology, Istanbul University Istanbul Faculty of Medicine, Istanbul, Turkey

**Keywords:** Robotic surgery, Lung ultrasound, Perioperative complications, Respiratory complications, Atelectasis

## Abstract

Robotic major abdominal surgeries are popular worldwide, yet very few clinical studies have investigated the effects of robotic surgery setup on respiratory outcomes. In this prospective observational study, it is aimed to demonstrate the change in ultrasonographic condition of the lungs throughout the robotic surgery and its relation with respiratory outcomes. Robotic radical prostatectomy patients without any preexisting lung or cardiac pathology were enrolled in the study. Lung ultrasound score (LUS) was chosen to evaluate lungs perioperatively in three different time points that is as follows: 5 min after intubation (T1), before extubation (T2), and 30 min after extubation (T3). Blood gas analyses were made at the same time points. Primary outcome was the change of LUS comparing T3 versus T1. Secondary outcomes included intraoperative change of LUS, severe postoperative pulmonary complication incidence, unplanned intensive care unit (ICU) admission incidence, comparison of oxygenation via PaO_2_ and PaO_2_/FiO_2_, and sensitivity/specificity of LUS for determining ICU admission. Total 48 patients were analyzed. T3 LUS was significantly higher than T1 LUS, and T2 was the highest amongst (15.5 [6, 25] vs 8.5 [4, 20] vs 20.5 [13, 30], respectively, *p* < 0.01). Pre-extubation LUS were significantly higher in patients who were admitted to ICU comparing who were not (23.5 [17, 30] vs 20 [13, 27], *p* = 0.03). PaO_2_/FiO_2_ ratio did not change among the groups significantly (*p* = 0.14). ROC curve of T2LUS showed 67% sensitivity and 85% specificity with a cut-off value of 22.5 for ICU admission (AUC 0.73 [0.516, 0.937], *p* = 0.04). LUS significantly worsened in robotic prostatectomy throughout the surgery, yet clinical deoxygenation or severe PPC were not observed. On the other hand, pre-extubation LUS may be used to determine possible ICU admission for the patients.

Clinical trial registry: This study was prospectively registered at Clinicaltrials.gov (NCT05528159).

## Introduction

Technological innovations in medicine open newer paths for everyday surgical practice. As laparoscopic surgeries have become “mainstream” in many disciplines, the search for improving minimal invasive techniques via reliable and precise methods is ongoing. Perhaps, one of the biggest progressions in modern medicine is integrating robotic systems into surgical setup. Currently, these robotic systems are used in a big variety of operations including urology, gynecology, general surgery, head and neck surgery, and cardiothoracic surgery [1]. A remote console allows the operator to manipulate several robotic arms which are proceeded in the surgical field beforehand, and the most important advantage is claimed to be reducing the number or even eliminating the operating physicians without compromising surgical precision [2].

Even though the surgical success appears to be increasing, general principles in minimally invasive techniques that are operating through several trocars, insufflation of carbon dioxide into the abdomen and Trendelenburg position are still valid for urological robotic surgery [3]. Physiological consequences of such manipulations are yet to be investigated under clinical terms. One might expect a prominent change in lung mechanics considering the position and intraabdominal insufflation. Ultrasound may allow instant evaluation of the lungs on the bedside. Despite the broad use of ultrasonography in many disciplines, “intraoperative” use of ultrasound for such purposes is not well investigated.

Recently developed lung ultrasound score (LUS) is a lung zone-based numeric assessment which shows aeration loss quantitatively in both hemithoraxes [4]. Performing this scoring system intraoperatively would provide a better clinical understanding in terms of respiratory mechanics and oxygenation. Therefore, in this prospective observational study, it is aimed to investigate the LUS changes in robotic radical prostatectomy surgeries throughout the perioperative process. A deterioration in LUS due to surgical manipulation was hypothesised. The primary outcome was comparison of the LUS which were assessed on 5th minute of intubation and 30th minutes of extubation. Secondary outcomes included intraoperative LUS change (difference between 5th minute of intubation and pre-extubation), severe postoperative pulmonary complications (defined as; hemothorax, pneumothorax, need for positive pressure ventilation and bronchospasm), unplanned intensive care unit (ICU) admissions, and intraoperative PaO_2_/FiO_2_ ratios.

## Methods

Following the local ethics committee approval (Istanbul University Istanbul Faculty of Medicine Clinical Research Ethics Committee-2022/1056), this trial was registered prospectively to clinicaltrials.gov (NCT05528159). Adult patients with American Society of Anesthesiologists Physical Status (ASA) I to II, who were scheduled for robotic radical prostatectomy surgery and provided consent, were included in the study between December 2022 and April 2024. Exclusion criteria included pre-existing chronic obstructive pulmonary disease requiring inhaled therapy, severe heart failure (patients with ejection fraction lower than 40%), pulmonary hypertension (Pulmonary artery pressure higher than 40 mmHg), and increased intracranial hypertension syndrome.

### Trial design

In this prospectively designed observational investigation, lung ultrasound was planned to be performed on three different time points in the perioperative period. Accordingly, LUS was assessed 5 min after intubation (baseline-T1), just before the extubation (T2) and 30 min after extubation (postoperative-T3). These assessments were executed by two experienced physicians (MSK, ESB) simultaneously to reduce possible bias related to the subjective nature of LUS.

### Perioperative anesthesia management

**Fig. 1:**
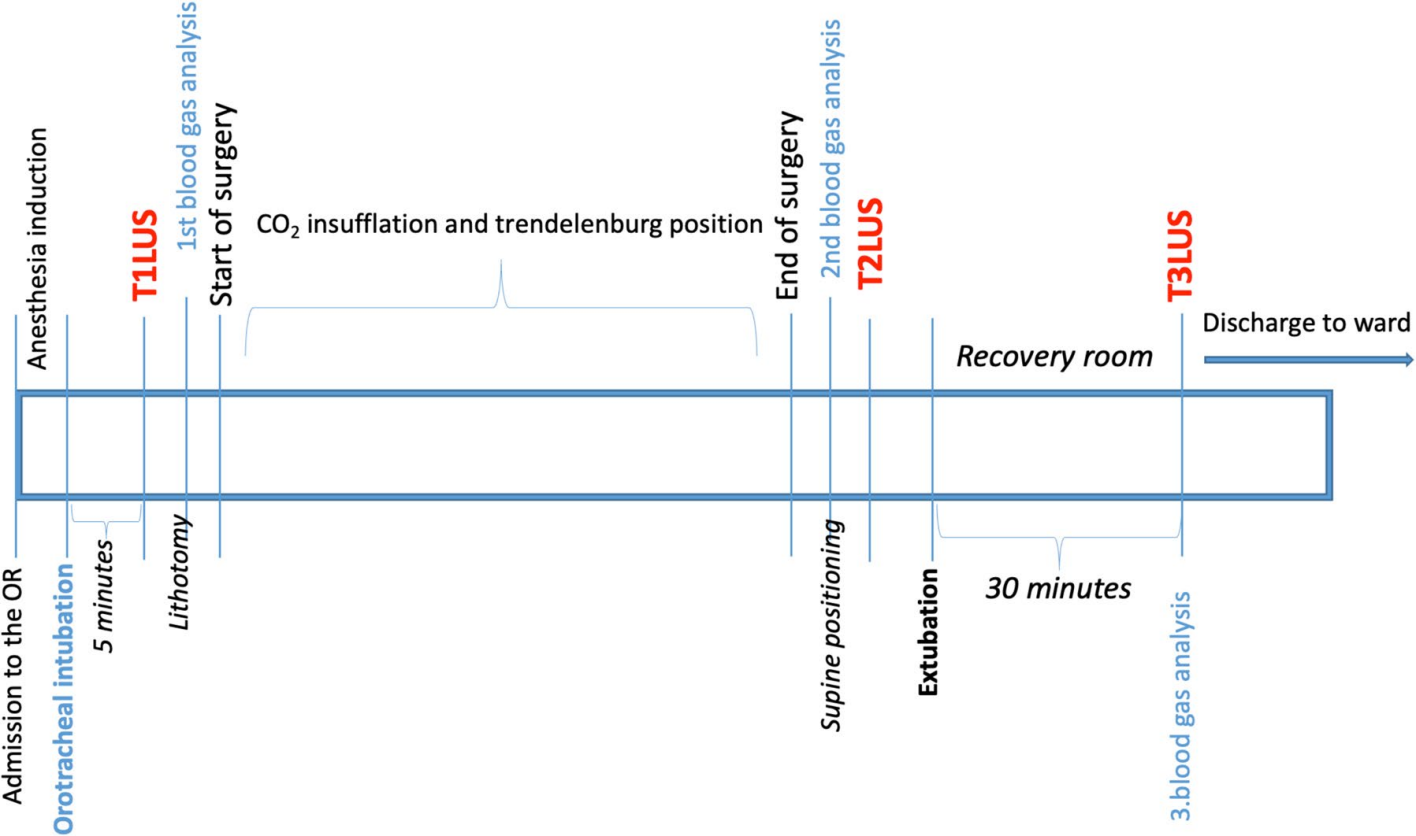
Study follow-up scheme

Standardized advanced monitorisation (pulse oximetry, electrocardiogram, invasive arterial blood pressure, capnograph, temperature and bispectral index) and intravenous (IV) general anesthesia induction with midazolam (0.05 mg/kg), fentanyl (2 mcg/kg), propofol (2–5 mg/kg) and rocuronium (0.6 mg/kg) were performed for all patients. Once safe intubation was provided, mechanical ventilation parameters were set as follows: positive end-expiratory pressure (PEEP) of 5 mmHg, tidal volume of 6–8 ml/kg, respiratory rate of 12–16/min. 40% of FiO_2_ with oxygen/air mixture was commenced to all patients, and N_2_O was avoided. Anesthesia was maintained with sevoflurane inhalation, which was set to keep minimum alveolar concentration at 1.2, and IV remifentanil infusion with a dose of between 0.05 and 0.2 mcg/kg/min. Baseline LUS (T1) was evaluated on the 5th minute after intubation. Next, the surgery was initiated, and accordingly, after introducing trochars and robotic arms, pneumoperitoneum was achieved via intraabdominal carbon dioxide insufflation and the patient were taken into 30º Trendelenburg position. After completion of the surgery and surgical closure, patient was positioned supine and T2 LUS was assessed. On the later period, safe extubation was provided, and the extubation was attempted in the operating room. The last LUS (T3) was assessed in Postanesthesia Care Unit (PACU) or ICU 30 min after extubation. Of note, all LUS assessments were made in supine position. The study follow-up is summarized in Fig. 1.

### Performing lung ultrasound score

**Fig. 2:**
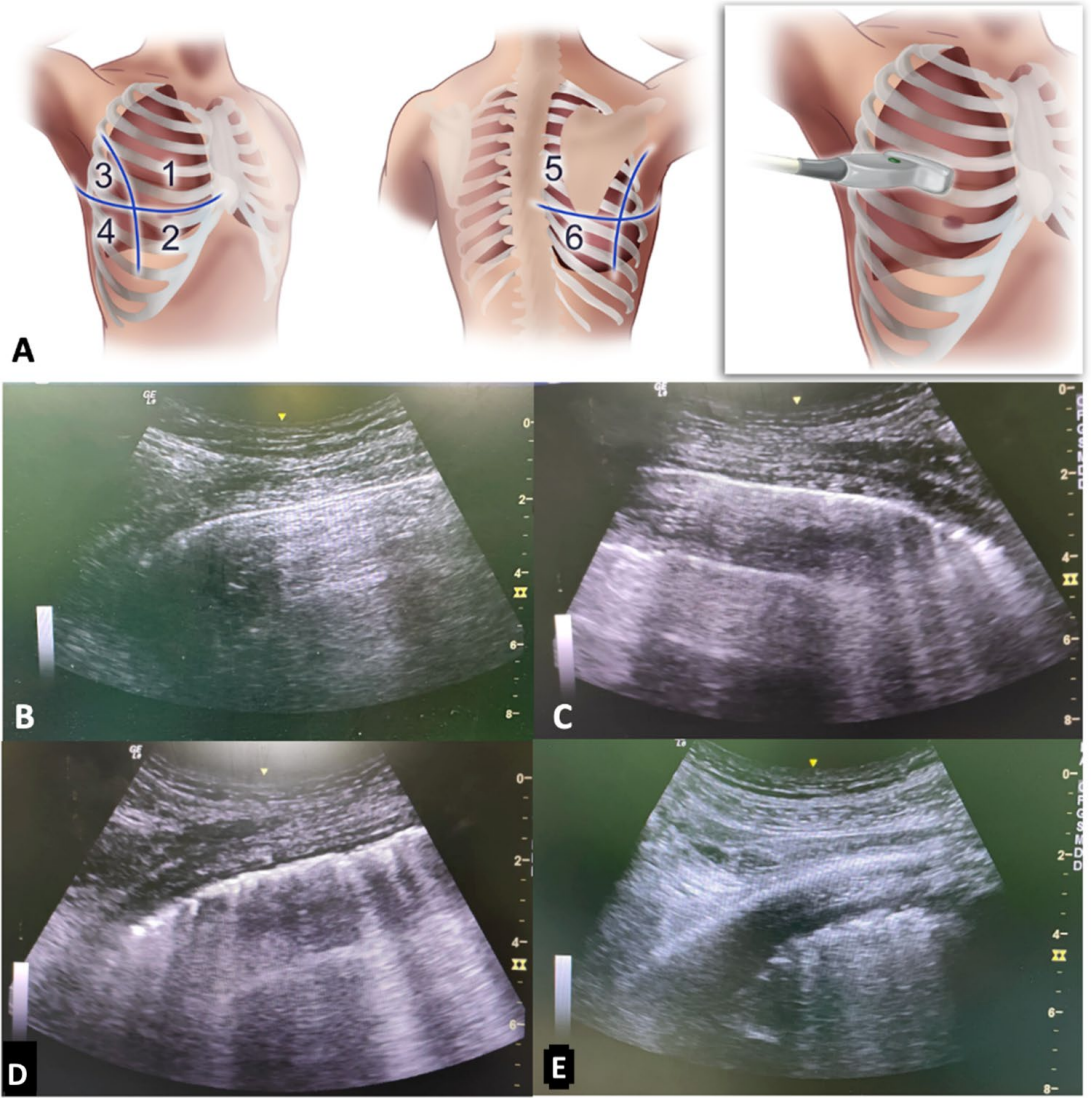
Illustration of how to perform LUS and ultrasonographic examples to scoring. **A** Assessment areas of the lungs. **B** Sole existence of physiologic transvers A-lines refers to 0 points, **C** existence of less than four pathologic horizontal B-lines refers to 1 point (mild loss of aeration), **D** existence of more than three B-lines or coalesced B-lines refer to 2 points (moderate to severe aeration loss), **E** existence of lung condensation with a separation of pleural sheets refers to 3 points (severe aeration loss)

The lungs were virtually divided into 6 quadrants per hemithorax via a horizontal line on the level of nipples, and sagittal lines on anterior axillary and posterior axillary. A convex ultrasound probe (2–5 MHz, GE LOGIQ™, Milwaukee, USA) was placed onto each quadrant (Fig. 2A) to observe pathologic signs and scoring was made as follows: sole existence of physiologic transvers A-lines refers to 0 points (Fig. 2B), existence of less than four pathologic horizontal B-lines refers to 1 point (mild loss of aeration) (Fig. 2C), existence of more than three B-lines or coalesced B-lines refer to 2 points (moderate to severe aeration loss) (Fig. 2D), existence of lung condensation with a separation of pleural sheets refers to 3 points (severe aeration loss) (Fig. 2E). Increasing scores exhibit worse aeration, and total 36 points for 12 different quadrants is possible.

### Outcome measures

The difference in lung ultrasound scores due to intraoperative period was analyzed. Along with the LUS, blood gas analysis were obtained at the same time points. PaO_2_/FiO_2_ ratios and blood gas parameters (pH, PaO_2_, PaCO_2_, SpO_2_, Base excess, and lactate) were documented. Additionally, unplanned ICU admissions and severe postoperative pulmonary complications (PPCs) were recorded. Severe PPCs included hemothorax (existence of blood in the pleural site verified by chest X-ray), pneumothorax (existence of air in the pleural site verified by chest X-ray), bronchospasm (existence of rhonchi requiring inhaled therapy), need for positive pressure ventilation (SpO_2_ drop below 92% despite adequate mask oxygenation). Lastly, the patients were also analyzed based on unplanned admittance to the ICU in terms of LUS, demographic and surgical data. The indications for unanticipated ICU admittance were defined as partial oxygen pressure lower than 60 mmHg in room-air and prolonged anesthesia duration exceeding 6 h.

### Statistical ANALYSES

Based on a study by Monastesse et al. [5] assuming a 30% increase in T3 LUS when compared to T1 (5 ± 2 vs 6.5 ± 3), while alpha error was 0.05 and with a power of 90%, necessity of 45 patients per group were calculated for statistical significance. Considering a possible drop-out of 10%, total 50 patients were planned to be enrolled in the study.

Chi-square test was used for categorical data analyses. Timely changes of LUS and blood gas parameters were evaluated via Friedman test. To observe the relation between LUS and unplanned ICU admission, ROC analyses were performed for sensitivity and specificity. When patients were divided according to ICU admission, between group analyses were provided via Mann–Whitney *U* test. Data were presented as mean ± standard deviation or median (min, max).

## Results

**Fig. 3:**
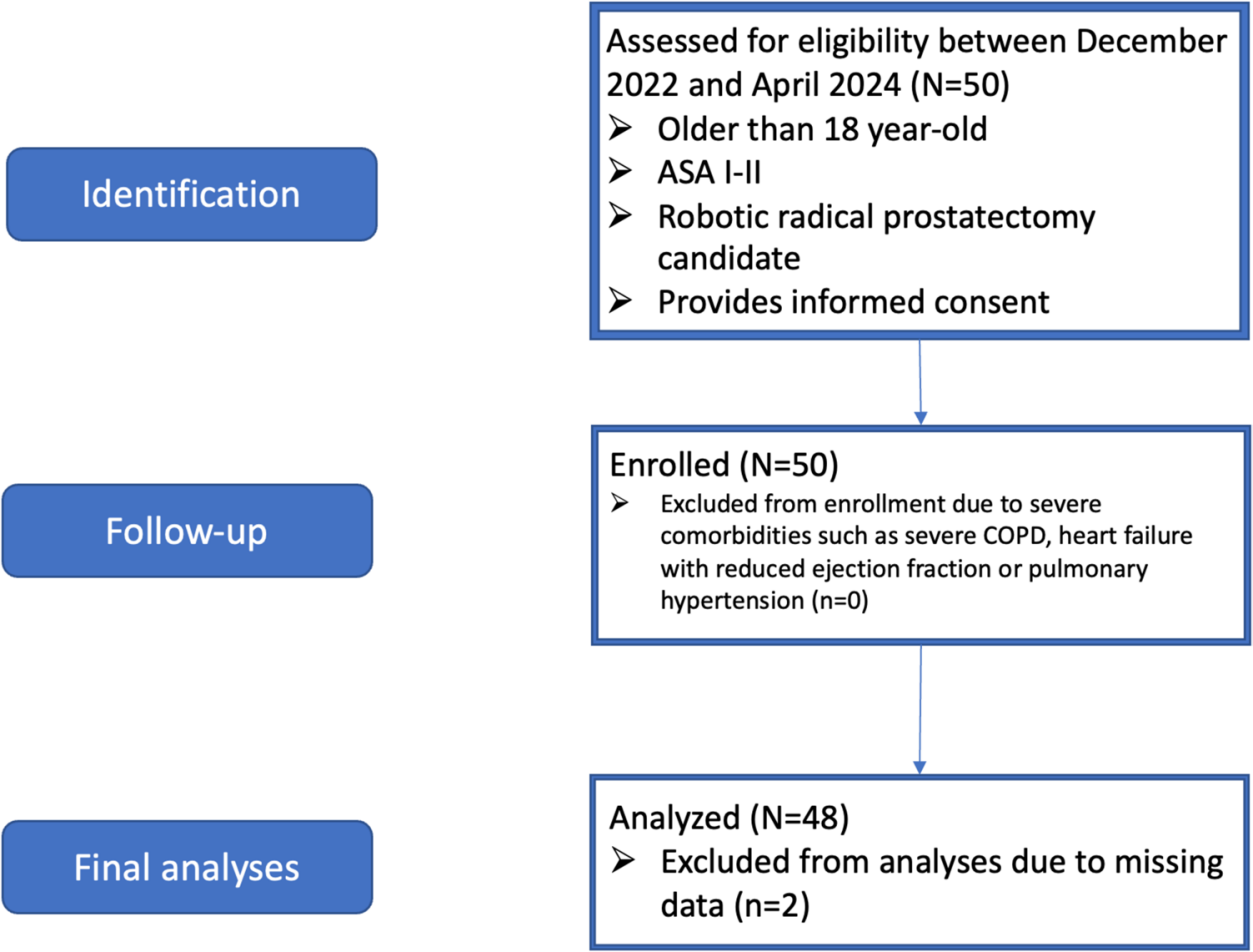
Study flowchart

50 patients were enrolled in the study between December 2022 and April 2024. Two of the patients were excluded from the final analyses due to missing data (Fig. 3). Demographic and surgical data are summarized in Table 1. Only one patient suffered from respiratory failure that required noninvasive positive pressure ventilation which was recorded as severe PPC, and total 9 patients were admitted to ICU postoperatively. Median length of stay was 4 (3, 10) days.

**Table 1:** Summary of demographic and surgical data

	Whole patient group (*N* = 48)
Age (years)	64.4 ± 7.9
BMI (kg/m^2^)	27.2 ± 3.5
ASA classification (*n*, %)	
ASA I	4, 8%
ASA II	44, 92%
Functional capacity (*n*, %)	
FC I	43, 89%
FC II	5, 11%
Comorbidities (*n*, %)	
Hypertension	25, 52%
Diabetes mellitus	14, 29%
Coronary artery disease	4, 8%
HF_p_EF	2, 4%
Atrial fibrillation	3, 6%
Active smoker	20, 42%
Anesthesia duration (min)	327.5 (220, 630)
Surgery duration (min)	292.5 (155, 560)
Unplanned ICU admission	9, 19%
Severe PPC	1, 2%
Length of stay in hospital (days)	4 (3, 10)

Data are presented as *n*, percentage; mean ± standard deviation; and median (min, max)

*BMI* body mass index, *ASA* American Society of Anesthesiologists Physical Status, *FC* functional capacity, *HF*_*p*_*EF* heart failure with preserved ejection fraction, *ICU* intensive care unit; *PPC* postoperative pulmonary complication

T3 LUS was significantly higher than T1 LUS (15.5 [6, 25] vs 8.5 [4, 20], *p* < 0.017), and T2 LUS was the highest with a median score of 20.5 (*p* < 0.017). This significance also applies for both sides, and also zone-based evaluation (*p* < 0.001) (Table 2). When unanticipated ICU admission was analyzed; pre-extubation LUS were significantly higher in patients who were admitted to ICU (23.5 [17, 30] vs 20 [13, 27], *p* = 0.03). Surgery and anesthesia duration were longer in patients admitted to ICU, either (380 ± 84 vs 277 ± 62 and 426 ± 90 vs 325 ± 65, respectively, *p* < 0.001). BMI were also higher in patients admitted to ICU (29.5 ± 4.6 vs 26.7 ± 2.9, *p* = 0.02) (Table 2).

**Table 2:** Time point based lung ultrasound score data along with demographic features of patients admitted to intensive care unit

	T1LUS	T2LUS	T3LUS	*p*
Right lung				
Upper part (zone 1 + 3 + 5)	2 (0, 5)	5 (2, 7)	4 (1, 6)	< 0.001^a^
Lower part (zone 2 + 4 + 6)	3 (0, 5)	6 (3, 8)	5 (1, 9)	< 0.001^a^
Posterior (zone 5 + 6)	2 (0, 4)	4 (2, 6)	3 (0, 5)	< 0.001^a^
Total	4.5 (1, 10)	11 (7, 15)	8 (4, 13)	< 0.001^a^
Left lung				
Upper part (zone 1 + 3 + 5)	1 (0, 6)	5 (1, 7)	3 (0, 6)	< 0.001^a^
Lower part (zone 2 + 4 + 6)	3 (0, 5)	5 (3, 8)	4 (1, 7)	< 0.001^a^
Posterior (zone 5 + 6)	2 (0, 5)	4 (2, 6)	3 (0, 9)	< 0.001^a^
Total	4 (1, 10)	10 (6, 15)	8 (2, 12)	< 0.001^a^
Both lungs	8.5 (4, 20)	20.5 (13, 30)	15.5 (6, 25)	< 0.001^a.b^
PaO_2_/FiO_2_ ratio	366.5 (183, 637)	349 (162, 536)	378 (134, 445)	0.20

	Patient admitted to ICU (*n* = 9)	ICU (–) (*N* = 39)	*p*
T2LUS (both lungs)	23.5 (17, 30)	20 (13, 27)	0.03
ASA classification			0.3
ASA I	0, 0%	4, 10%	
ASA II	9, 100%	35, 90%	
BMI	29.5 ± 4.6	26.7 ± 2.9	0.02
Anesthesia duration (min)	426 ± 90	325 ± 65	< 0.001
Surgery duration (min)	380 ± 84	277 ± 62	< 0.001

Data are presented as median (min, max); number, percentage (%); and mean ± standard deviation

*T1LUS* 5 min after intubation lung ultrasound score, *T2LUS* pre-extubation lung ultrasound score, *T3LUS* 30 min after extubation lung ultrasound score, *ASA* American Society of Anesthesiologists Physical Status, *BMI* body mass index

^a^T1 is significantly lower than T2 and T3

^b^T2 is significantly higher than T1 and T3

PaO_2_ were significantly lower in T3 time-point blood gas analysis (90 [56.4, 174] vs 166 [88.6, 525] in T1 vs 163 [58, 536] in T2, *p* < 0.001), yet PaO_2_/FiO_2_ ratio did not change among the groups significantly (*p* = 0.14) (Tables 2, 3).

**Table 3:** Blood gas data presented for different time points

Blood gas parameter	T1	T2	T3	*p*
pH	7.38 (7.24, 7.50)	7.37 (7.28, 7.50)	7.38 (7.31, 7.48)	0.6
PaO_2_ (mmHg)	166 (88.6, 525)	163 (58, 536)	90 (56.4, 176)	< 0.001
PaCO_2_ (mmHg)	42 (30, 53)	40.9 (32.5, 51.4)	38.8 (31.1, 55)	0.01
SpO_2_ (%)	99 (96, 100)	99 (88, 99.9)	97 (81, 100)	< 0.001
Base excess (mmol/l)	0.05 (− 6.2, 8.6)	− 1.3 (− 6, 8.2)	− 1.6 (− 6.2, 7)	< 0.001
Lactate (mmol/l)	1.15 (0.3, 3.1)	1.3 (0.6, 3.4)	2 (0.9, 6.4)	0.14

Data are presented as median (min, max)

**Fig. 4:**
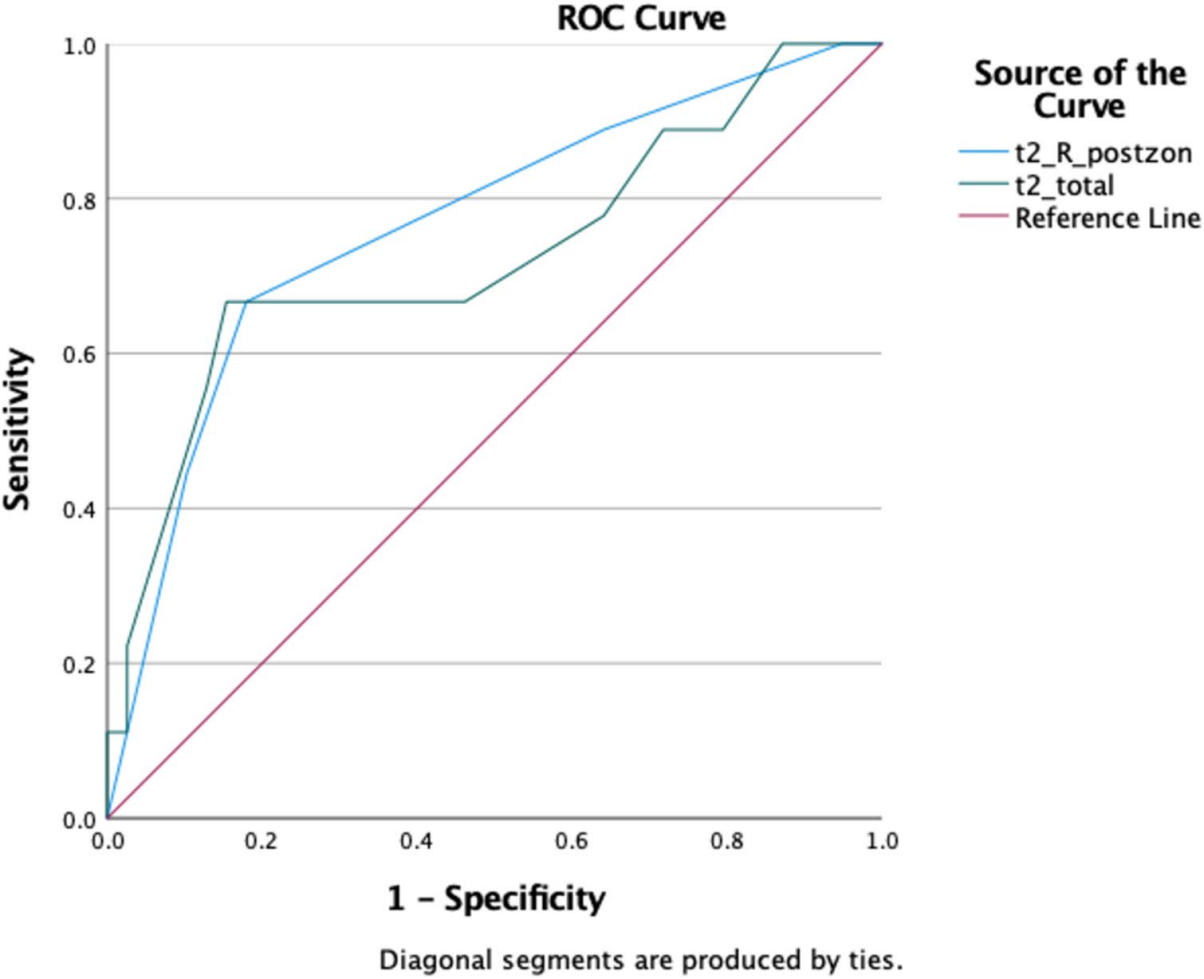
ROC analyses of total LUS and right lung posterior zone LUS at T2 time point. T2-R-Postzon: AUC: 0.766 (0.585, 0.948), p:0.014*, sensitivity: 0.667 specificity: 0.821, cutoff: 4.5. T2-total: AUC: 0.726 (0.516, 0.937), *p*: 0.036*, sensitivity: 0.667 specificity: 0.846, cutoff: 22.5

ROC curve of T2LUS for ICU admission showed 67% sensitivity and 85% specificity with a cut-off value of 22.5 (AUC 0.73 [0.516, 0.937], *p* = 0.04). Similarly, summation of right lung posterior zone (zone 5 + 6) LUS showed 67% sensitivity and 82% specificity (AUC 0.77 [0.585, 0.948] cut-off:4.5, *p* = 0.014) for ICU admission (Fig. 4).

## Discussion

In this pragmatic, prospective, observational study, it was aimed to demonstrate possible respiratory manifestations of robotic major urologic surgery via interpreting lung ultrasound findings, and its clinical reflections. Accordingly, a distinctive (and ultrasonographic) aeration loss is observed with “robotic surgery”, peaking before extubation and diminishing in the early postoperative period, yet it does not translate into hypoxemia or severe pulmonary complication.

As an advancement to minimal invasive techniques, robotic surgery has become marginally popular in many surgical disciplines. Despite the positive “surgical” outcomes, “anesthetic” or “systemic recovery” outcomes may not be enhanced [6]. Respiratory complications are the occult threat in the postoperative period from anesthetic perspective, and literature regarding the “intraoperative” use of ultrasound to observe lungs’ condition are very sparse. Vast majority of those are focused finding the right ventilatory strategy rather than demonstrating surgery based effects on the lungs. The only study looking into “perioperative use of lung ultrasound” was by Szabo et al. [7]. They suggested using lung ultrasound in postoperative 24th hour to foresee respiratory complications, and that is not surprising since PPCs refer to the respiratory complications in postoperative 7 days [8].

Pneumoperitoneum is known to be causing atelectasis at some extent. Theoretically, after carbon dioxide insufflation into the peritoneum, diaphragm is pushed superiorly which causes involution in lower parts of the lungs. Since this is a common and well-accepted knowledge, very few trials have used ultrasonography perioperatively to evaluate lungs under definitive manners, and most of them were conducted in only “laparoscopic” or “open” surgeries. Nevertheless, the existing findings are solid. Genereux et al*.* have demonstrated deteriorated Lung Ultrasound Score in open gynecologic surgery, despite the use of lung protective ventilation modalities (mean increase in LUS was calculated to be 2.4 points) [9]. Similar deterioration was also shown in a study by Fu et al. in which median LUS was increased from zero to five points in abdominal laparotomy patients [10] Moderate pulmonary risk group patients were enrolled in this study, and lung protective ventilation was compared against conventional method. Accordingly, PPC incidence and aeration loss were lower in group with protective ventilation. In the light of these data, use of lung protective mechanical ventilation methods with frequent recruitment maneuvers, 6–8 mmHg of positive end-expiratory pressure, and 6–8 ml of tidal volume may be suggested for robotic abdominal surgeries, either. Similar modality was chosen for our investigation except for frequent recruitment maneuver. Performing recruitment maneuver would interfere with our results since our aim was to demonstrate to what extent robotic surgery would affect the lungs. Considering these, one may draw attention to the extensive increase of LUS in our study which reflects a rise from 8.5 to 20.5 points intraoperatively, and then a drop to 15.5 points. Further studies may be planned to observe the actual effect of recruitment maneuvers on intraoperative LUS scores in robotic surgeries which has the possibility of demonstrating an amelioration. Unfortunately, our study design was not built for this particular aim.

One may emphasize the “pulmonary risk” of the patient population. The loss of aeration has always been a matter of concern in anesthesiology practice, yet this reduction in the aeration may not necessarily become a respiratory complication. For example, Park et al. have successfully managed to exhibit a reduction in atelectasis when ultrasound guided recruitment maneuver is performed to the lungs, also showing a different way of perioperative ultrasound usage [11]. However, a difference in PPC outcome was not observed possibly due to “low risk” of the patients in this study. On the other hand Fu et al. were successful to demonstrate significant difference with the incidence of PPC with their “moderate-risk” patients [10]. In the current study, we bring a different perspective to use of LUS for determining patients who might require intensive care unit admission. As noted, pre-extubation LUS was significantly higher in patients who were admitted to ICU postoperatively which might be due to prolonged procedure (both anesthesia and surgery), and ROC analyses have demonstrated a great specificity (85%) for the cut-off value of 22.5. Accordingly, patients with a pre-extubation LUS lower than 23 might be followed up in ward safely after the surgery. Therefore, this cut-off (which still needs validating via extensive clinical trials) may serve as a reliable limit to determine if the patient needs postoperative ICU follow-up.

The only clinical investigations in “robotic surgery” population are by Yoon et al. and Lee et al*.* [12, 13]. Both of these studies were randomized trials investigating two different mechanical ventilation methods, yet they showed a prominent increase in LUS during the surgery. Compatible with our findings, there is a certain pattern visible within all abovementioned studies which is an evident LUS increase from beginning to the end of surgery and a clear decrease in the acute postoperative period [5, 9, 10]. This may be due to the diaphragm working effectively after curarization completely diminishes. In the current study, even though the LUS have changed drastically, oxygenation was within acceptable range except one patient requiring non-invasive mechanical ventilation.

Many recent literature claim the superiority of ultrasound over other conventional assessment tools such as chest X-ray or auscultation [14–16]. As authors of this manuscript, we also encourage the perioperative use of ultrasound since it is a bed-side, easy-to-use and quick imaging technique. Considering these features, physicians may have the chance to intervene faster or even they may prevent the complication before occurring. No doubt, being able to understand the ongoing pathology would be first the step. Therefore, more clinical studies are needed. Our results have provided a small insight regarding the unanticipated ICU admission when a cut-off LUS value of 22.5 is exceeded right before extubation. This suggestion is debatable since sensitivity is moderate (67%). On the other hand, high specificity (85%) may be interpreted as a safety margin expecting a safer postoperative period for those with LUS lower than 22.5. Our results also revealed that evaluating right lung posterior zone would provide a similar benefit. Perhaps, larger clinical studies would help defining those thresholds more accurately, and this would give a hint about which patient to develop pulmonary complications, also. Of note, ultrasound is a physician-dependent, subjective assessment which we tried to overcome via two-person consensus during the measurements. Therefore, subjectivity and profession-requiring nature of ultrasonography would counted as the limitation of the current study. On the other hand, the best way to use LUS would be for identifying patients who are prone to develop PPCs after surgery. However, the current study did not provide necessary number of participants to define a certain cut-off value for increased risk of PPC. Therefore, limited participant number would be is another limitation.

## Conclusion

Major urologic robotic surgery leads aeration loss and partial atelectasis in the lungs which do not alter oxygenation and turn into a pulmonary complication in the postoperative period. Ultrasonography stands as a powerful tool to diagnose such changes. Comparative studies with regard to lung ultrasound between different surgical techniques (open vs laparoscopic vs robotic) might be conducted to enlighten which technique causes the aeration loss most. Therefore, further clinical studies with larger participant numbers are needed.

## Data Availability

The clinical data of this trial is available upon reasonable request to the corresponding author. No datasets were generated or analysed during the current study.
